# A Simple and Effective Method for Endoscopic Fragmentation of Gastric Bezoars 

**DOI:** 10.5152/tjg.2026.26208

**Published:** 2026-07-08

**Authors:** Haijun Liang, Tao Yin, Zhenyu Liu

**Affiliations:** The 96605 Military Hospital, Tonghua, China

## Case Presentation

**Figure 1.**
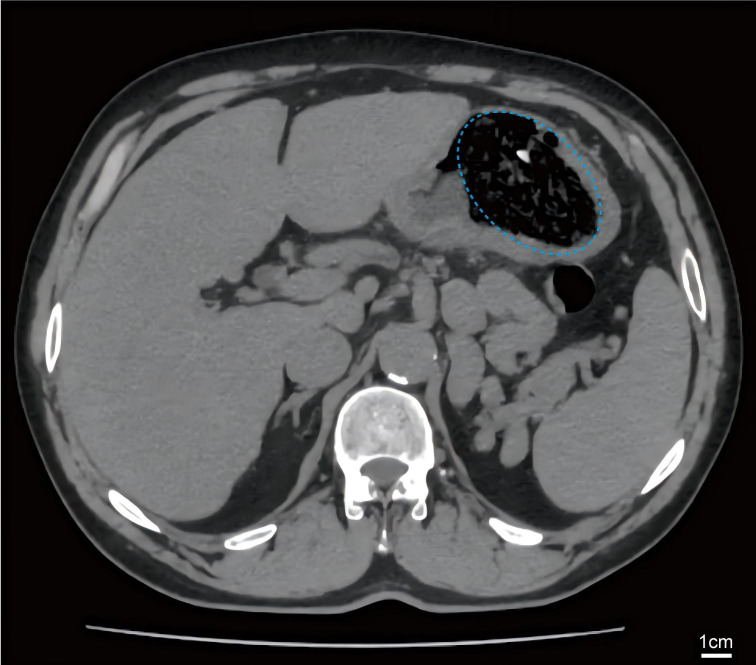
Computed tomography revealed a giant gastric bezoar.

**Figure 2.**
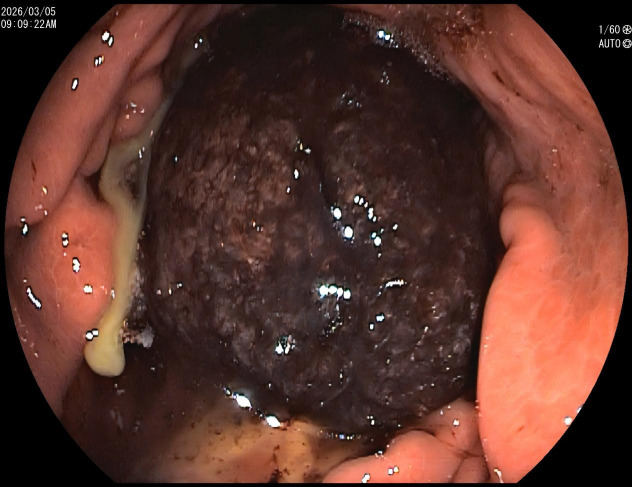
Gastric bezoar before treatment.

**Figure 3.**
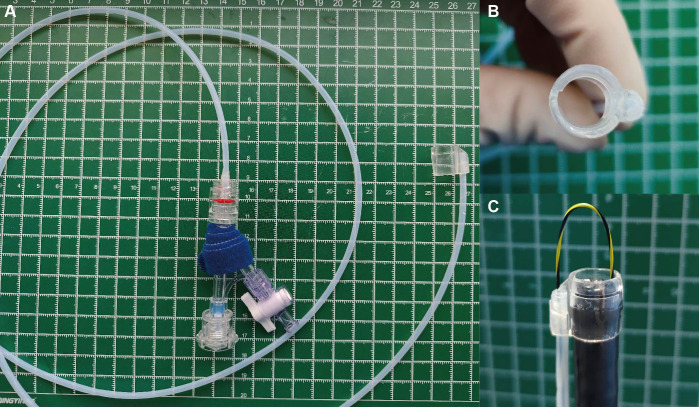
Device used in this study. (A) Endoscopy-assisted channel catheter. (B) Tip of the device. (C) Device with the guidewire loaded onto the endoscope.

A 60-year-old man presented with gastric pain and nausea that developed after consuming hawthorn-containing foods for more than 1 month. Computed tomography revealed a giant gastric bezoar measuring 6.7 × 3.7 cm (Figure 1). On gastroscopy, the gastric bezoar appeared hard with a smooth surface and dark brown coloration (Figure 2). The patient received 10 mL of 5% NaHCO_3_ orally 3 times a day, for 5 days. After the bezoar had softened, endoscopic bezoar fragmentation was performed using an endoscopy-assisted channel catheter (Micro-Tech [Nanjing] Co., Nanjing, China) and a yellow zebra guidewire (Figure 3). Verbal informed consent was obtained from the patient before participation in the study. No generative AI was used during the preparation of the manuscript.

## Technique

**Figure 4:**
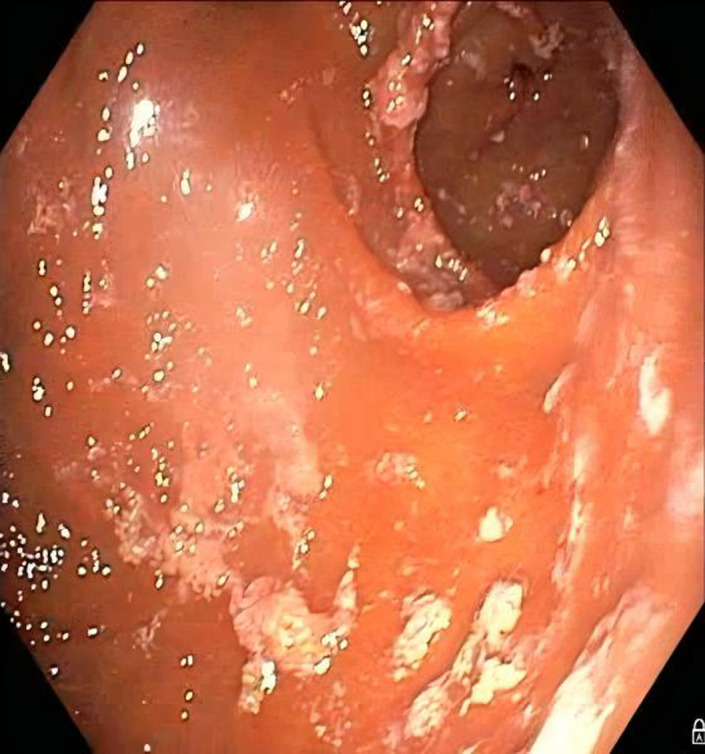
Follow-up image of this patient obtained 3 days after treatment, showing complete clearance of the gastric bezoars from the stomach.

With the catheter mounted on the endoscope, the guidewire was passed through the biopsy channel and then passed retrogradely through the catheter. The preparation process took approximately 2 minutes. The endoscope was inserted into the stomach. Although the surface of the bezoar had softened, it remained a large mass. The assistant secured one end of the guidewire while the operator manipulated the other end to form a loop around the bezoar. The operator tightened the guidewire loop to snare and fragment the bezoar. This procedure was repeated until the bezoar was reduced to small fragments (Video 1). Fragmentation required approximately 8 minutes. The patient’s symptoms improved substantially. After 3 days of treatment with 5% NaHCO_3_, a follow-up gastroscopy confirmed complete clearance of bezoars from the stomach (Figure 4).

## Discussion

Endoscopic fragmentation is a safe and effective treatment method for gastric bezoars. Giant gastric phytobezoars remain a significant challenge because of their large size, hardness, and insolubility. Standard nonsurgical approaches, such as snares and lithotripters, have limitations such as prolonged procedure times and the risk of incomplete removal.^1^ Guidewire-assisted fragmentation has been shown to be effective for the treatment of bezoars. However, because the guidewire must be folded to pass through a single channel, only guidewires measuring 0.025 inches or less in diameter can be used with a standard endoscope.^2^ A “hand-made bezoaratome” has been reported to be safe and effective for the treatment of large bezoars; however, guidewire diameter remains limited. Reproducibility across centers may also be limited because the device is not standardized.^3^ A novel guidewire-based device has also been used for the treatment of giant bezoars, but its outer diameter is increased by the attached sheaths, which may increase the risk of mucosal injury during insertion.^4^ In the current case, n endoscopy-assisted channel catheter and a guidewire were used for the successful fragmentation of giant bezoars. The catheter features a beveled tip for easier insertion without injury to the mucosa. The device is standardized and easy to install and use. It can also provide an additional channel for the endoscope, thereby enabling a broader range of endoscopic treatments in almost every endoscopy center.

## Conclusion

The current case report demonstrates the safety and effectiveness of this novel technique for the treatment of large gastric bezoars. Because of its excellent ease of use, it holds broad application prospects in resource-limited settings.

## Data Availability

The data that support the findings of this study are available on request from the corresponding author.
